# Supplementary material for: Suicide-Related Mortality Trends in Europe, 2012–2021
Source: Int J Environ Res Public Health. 2025 Jun 2;22(6):890. doi: 10.3390/ijerph22060890 (PMC12193659; doi:10.3390/ijerph22060890)
Supplement: Supplementary file 1 [file ijerph-22-00890-s001.zip › ijerph-3552795-supplementary.pdf]

Supplementary Materials

Table S1. Suicide-related age-adjusted mortality in different European countries, 2012–2020.

	2012	2013	2014	2015	2016	2017	2018	2019	2020	2021	AAPC	95% CI p	Joinpoints	APC Period 1 [Years]; APC (95% CI)p	APC Period 2 [Years]; APC (95% CI)p
Belgium	18,78	17,3	17,29	16,85	17,09	15,44	16,01	15,28	15,27	14,34	-2.2	-2.7 to -1.8; <0.001	0	-	-
Bulgaria	12,11	9,78	9,91	9,34	9,23	9,78	8,83	8,01	8,04	7,82	-3.4	-4.7 to -2.2 <0.001	0	-	-
Czechia	15,97	15,18	14,36	13,25	12,55	13,17	12,82	11,22	11,64	11,64	-3.1	-5.2 to -1.3 <0.001	0	-	-
Denmark	12,22	11,28	11,91	10,21	10,19	10,45	10,34	10,57	10,03	9,46	-1.7	-3.2 to -0.3 0.01	0	-	-
Germany	11,77	11,9	11,94	11,68	11,3	10,54	10,61	10,18	10,31	10,28	-1.9	-2.4 to -1.4 <0.001	0	-	-
Estonia	18,4	16,96	18,31	15,89	14,31	17,22	14,85	14,89	16,28	14,01	-1.8	-4.1 to 0.6 0.13	0	-	-
Ireland	12,12	11,07	10,79	9,49	9,37	8,38	9,41	8,17	9,62	9,31	-2.9	-4.4 to -1.5 <0.001	0	-	-
Greece	4,43	4,77	4,99	4,69	4,29	4,52	4,93	4,63	4,03	4,15	-0.1	-1.8 to 1.4 0.80	0	-	-
Spain	7,43	8,14	8,17	7,51	7,41	7,51	7,18	7,39	7,89	7,93	1.0	-1.4 to 2.9 0.24	0	-	-
France	15,71	15,48	14,09	14,12	13,2	12,76	13,48	13,36	13,51	13,32	-2.2	-2.8 to -1.5 <0.001	0	-	-
Croatia	18,23	16,18	16,84	17,32	16	14,8	15,96	14,01	13,19	14,06	-2.5	-4.4 to -0.6 0.01	0	-	-
Italy	6,68	6,64	6,32	6,07	5,85	5,95	5,71	5,62	5,61	5,89	-1.7	-2.5 to -1.0 <0.001	0	-	-
Cyprus	3,82	5,19	4,5	4,45	3,89	4,06	4,57	4,35	3,45	2,68	-4.4	-7.3 to -0.9 0.02	0	-	-
Latvia	21,82	19,05	18,96	19,34	18,67	17,98	15,57	15,04	15,59	13,38	-4.3	-5.8 to -2.7 <0.001	0	-	-
Lithuania	30,69	36,12	31,51	30,28	28,17	26	23,98	22,89	21,29	19,51	-4.8	-5.6 to -3.9 <0.001	0	-	-
Luxembourg	10,69	9,29	13,36	13,91	9,38	9,38	9,01	11,52	9,99	6,12	-3.5	-9.7 to 2.9 0.23	0	-	-
Hungary	24,14	21,24	19,42	19,03	17,97	16,66	16,84	15,65	17,09	15,69	-4.2	-5.2 to -3.3 <0.001	0	-	-
Malta	6,4	5,02	8,15	7,39	5,33	5,23	4,11	3,95	3,98	6,59	-2.2	-10.7 to 7.1 0.54	0	-	-
Netherlands	10,67	11,32	11,12	11,28	11,29	11,22	10,69	10,48	10,49	10,57	0.5	-0.1 to 1.1	0	-	-

												0.07			
Austria	15,2	15,35	15,25	14,45	13,63	13,84	13,62	12,32	11,73	11,94	-2.8	-3.7 to -1.9<br><0.001	0	-	-
Poland	16,71	16,35	15,51	14,23	12,28	11,7	11,7	11,97	11,93	12,15	-3.9	-5.7 to -2.1<br><0.001	0	-	-
Portugal	9,98	9,78	11,28	10,38	8,95	9,61	9,03	8,76	8,48	8,22	-1.5	-2.7 to -0.4<br>0.006	0	-	-
Romania	12,67	12,24	11,33	11,43	10,16	9,97	10,03	9,04	9,04	9,33	-3.8	-4.6 to -3.0<br><0.001	0	-	-
Slovenia	21,53	21,7	18,87	20,72	18,09	19,52	16,86	18,23	17,06	19,81	-1.4	-4.3 to 0.8<br>0.15	0	-	-
Slovakia	11,12	12,07	10,74	9,19	7,48	7,23	7,94	6,97	6,9	7,5	-4.7	-7.7 to -2.5<br><0.001	0	-	-
Finland	16,14	16,36	14,55	13,46	14,26	15,01	14,4	13,47	12,95	13,48	-2.4	-3.3 to -1.3<br><0.001	0	-	-
Sweden	12,39	13,03	12,13	12,3	11,73	12,08	12,73	12,75	11,91	12,08	0.1	-1.0 to 1.0<br>0.97	0	-	-
Iceland	11,61	17,1	13,98	13,06	12,39	9,62	10,23	11,33	12,62	9,88	1.0	-2.3 to 3.8<br>0.57	0	-	-
Liechtenstein	9,64	7,48	10,22	12,49	15,59	14,14	19,99	25,1	15,48	14,91	-3.3	-10.2 to 19.5<br>0.64	0	-	-
Norway	10,62	11,25	10,97	11,71	12,1	11,6	12,93	12,38	12,02	12,11	0.5	-0.6 to 2.1<br>0.31	0	-	-
Switzerland	13,2	13,35	12,77	12,96	12,19	12,33	11,92	11,9	11,29	11,48	-1.5	-2.3 to -1.0<br><0.001	0	-	-
Serbia	17,28	16,81	15,88	15,03	13,52	14,24	13,34	13,26	12,67	12,56	-3.7	-4.2 to -3.1<br><0.001	0	-	-

AAMR: Age-adjusted mortality rate, expressed as deaths per 100.000 population. AAPC: Average Annual percent change; APC: Annual percent change.

Table S2. Suicide-related age-adjusted mortality in men of different European countries, 2012–2020.

	2012	2013	2014	2015	2016	2017	2018	2019	2020	2021	AAPC	95% CI p	Joinpoints	APC Period 1 [Years]; APC (95% CI)p	APC Period 2 [Years]; APC (95% CI)p
Belgium	28,96	26,09	25,7	24,69	25,5	23,05	23,97	23,28	23,18	21,34	-2.4	-2.9 to -2.0 <0.001	0	-	-
Bulgaria	20,86	17,1	16,94	15,74	15,84	17,07	15,13	14,01	14,14	13,59	-3.3	-4.5 to -2.0 <0.001	0	-	-
Czechia	28,64	26,84	24,61	23,12	21,76	22,19	22,16	19,04	20,71	19,75	-3.7	-5.1 to -2.2 <0.001	0	-	-
Denmark	19,05	17,59	18,37	14,62	15,82	15,65	15,62	16,59	14,93	14,09	-2.1	-3.6 to -0.6 0.005	0	-	-
Germany	18,96	19,18	19,33	18,62	18,21	17,16	17,25	16,45	16,62	16,29	-2.1	2.5 to -1.7 <0.001	0	-	-
Estonia	34,33	32,28	33,64	29,85	26,35	30,16	27,22	26,36	29,32	24,98	-2.4	-4.9 to 0.1 0.06	0	-	-
Ireland	20,44	17,99	17,95	15,21	15,23	13,83	14,46	12,84	14,18	15,15	-3.0	-4.3 to -2.1 <0.001	1	[2012-2014] -5.8 (-7.8 to -4.7) <0.001	[2014-2021] 8.9 (-0.1 to 14.3) 0.06
Greece	7,58	7,95	8,42	7,87	7,55	7,71	8,14	8,06	6,86	7,29	-0.1	-1.4 to 1.3 0.91	0	-	-
Spain	12,28	12,93	13,07	11,93	11,74	11,77	11,26	11,78	12,46	12,53	0.1	-1.5 to 1.8 0.88	0	-	-
France	25,79	25,33	22,97	23,42	21,95	20,77	21,99	21,85	21,89	21,71	-2.1	-2.7 to -1.5 <0.001	1	[2012-2017] -4.1 (-6.1 to -3.0) <0.001	[2017-2021] 1.0 (-1.0 to 5.2) 0.29
Croatia	31,58	28,25	28,25	29,01	27,1	24,88	27,03	25,67	22,89	24,82	-2.2	-3.8 to -0.5 0.009	0	-	-
Italy	11,2	11,02	10,54	10,16	9,82	9,89	9,32	9,29	9,39	9,73	-1.6	-2.2 to -1.2 <0.001	1	[2012-2019] -2.6 (-3.9 to -2.1) 0.003	[2019-2021] 2.6 (-1.3 to 4.7) 0.20
Cyprus	7,54	9,59	6,81	7,05	6,87	7,08	7,69	8,05	5,36	3,47	-7.2	-9.6 to -4.1 <0.001	0	-	-
Latvia	41,56	36,99	35,45	35,5	34,39	33,16	30,81	26,51	29,93	26,48	-4.4	-5.5 to -3.3 <0.001	0	-	-
Lithuania	56,59	66,31	59,02	56,25	54,24	47,37	44,38	42,82	38,1	35,22	-4.8	-5.8 to -3.8 <0.001	0	-	-
Luxembourg	17,08	12,17	21,62	20,69	14,23	14,3	12,5	17,08	16,66	9,43	-2.7	-11.8 to 7.5 0.48	0	-	-
Hungary	42,22	36,71	33,93	32,58	30,84	29,23	30,37	26,97	30,39	27,52	-4.2	-5.4 to -3.0 <0.001	0	-	-

Malta	11,13	9,63	16,01	11,43	8,92	8,55	7,33	7,2	5,06	9,09	-5.1	-9.6 to -0.3<br>0.03	0	-	-
Netherlands	14,92	16,48	15,56	16,01	15,75	15,67	14,18	14,69	14,51	15,04	0.2	-0.7 to 1.1<br>0.67	1	[2012-2013]<br>6.9<br>(1.2 to 12.2)<br>0.003	[2013-2021]<br>-1.4<br>(-3.1 to -0.7)<br>0.004
Austria	25,84	25,6	25,29	24,19	22,66	23,76	23,49	20,88	19,95	21,01	-2.6	-3.7 to -1.5<br><0.001	0	-	-
Poland	30,76	30,07	28,28	26,19	22,84	21,3	21,53	21,98	21,98	22,07	-3.8	-5.2 to -2.6<br><0.001	1	[2012-2017]<br>-6.2<br>(-12.7 to -4.0)<br>0.01	[2018-2021]<br>-0.1<br>(-4.1 to 8.4)<br>0.96 to 18.3
Portugal	18,03	16,94	19,23	16,75	15,44	16,12	15,13	15,04	14,9	14,17	-2.2	-3.3 to -1.1<br><0.001	0	-	-
Romania	22,98	21,78	20,49	20,55	18,17	17,52	17,85	16,48	16,64	17,15	-3.2	-4.3 to -2.5<br><0.001	0	-	-
Slovenia	39,57	37,14	33,44	36,15	30,7	34,71	28,56	30,8	29,7	33,29	-2.6	-4.0 to -1.1<br><0.001	0	-	-
Slovakia	21,08	22,94	20,34	16,66	13,69	13,06	13,85	12,78	13,16	13,64	-5.9	-8.8 to -3.0<br><0.001	0	-	-
Finland	25,29	25,41	22,79	21,26	22,98	22,89	22,6	20,88	19,55	19,53	-3.0	-4.1 to -1.7<br><0.001	0	-	-
Sweden	18,07	18,64	17,04	18,09	16,5	17,45	17,98	17,66	17,3	17,41	-0.2	-1.2 to 0.8<br>0.62	0	-	-
Iceland	16,71	24,68	21,49	18,23	21,66	17,57	16,83	17,65	16,21	12,89	-2.0	-7.5 to 3.9<br>0.44	0	-	-
Liechtenstein	10,12	14,92	20,38	15,05	10,26	28,4	35,07	34,7	22,59	10,13	2.8	-51.3 to 24.8<br>0.76	0	-	-
Norway	15,38	15,45	16,3	15,95	16,48	15,89	18,15	17,87	17,54	17,74	0.2	-0.7 to 1.9<br>0.55	0	-	-
Switzerland	20,55	21	19,89	20,34	19,18	19,28	18,13	18,3	16,95	17,23	-2.1	-2.8 to -.14<br><0.001	0	-	-
Serbia	28,6	28,22	26,43	24,76	21,77	23,8	22,18	21,43	21,15	20,93	-3.6	-4.6 to 2.6<br><0.001	0	-	-

AAMR: Age-adjusted mortality rate, expressed as deaths per 100.000 population. AAPC: Average Annual percent change; APC: Annual percent change.

Table S3. Suicide-related age-adjusted mortality in women of different European countries, 2012–2020.

	2012	2013	2014	2015	2016	2017	2018	2019	2020	2021	AAPC	95% CI<br>p	Joinpoints	APC Period 1 [Years]; APC (95% CI)p	APC Period 2 [Years]; APC (95% CI)p
Belgium	9,76	9,48	9,88	9,68	9,51	8,55	8,78	7,92	8,14	8,09	-2.8	-2.7 to -1.0<br><0.001	0	-	-
Bulgaria	5,1	3,75	4,24	4,02	4	4,15	3,8	3,13	3,07	3,21	-3.4	-6.9 to 0.3<br>0.67	0	-	-
Czechia	5,18	5,28	5,46	4,76	4,73	5,3	4,62	4,43	3,96	4,76	-1.6	-3.8 to 0.5<br>0.13	0	-	-
Denmark	5,9	5,73	5,89	6,11	4,96	5,64	5,48	5,15	5,61	5,11	-1.1	-2.5 to 0.3<br>0.12	0	-	-
Germany	5,82	5,85	5,74	5,93	5,37	4,92	4,93	4,72	4,85	5,09	-2.1	-3.6 to -0.6<br>0.006	0	-	-
Estonia	6,16	6,1	6,5	5,08	5,1	7,31	5,8	5,62	6,31	5,2	-0.2	-3.9 to 3.6<br>0.86	0	-	-
Ireland	4,08	4,28	3,82	3,93	3,65	3,2	4,48	3,63	5,2	3,64	0.2	-3.3 to 3.8<br>0.92	0	-	-
Greece	1,56	1,87	1,92	1,84	1,41	1,66	2,08	1,55	1,51	1,33	-0.9	-6.4 to 4.9<br>0.65	0	-	-
Spain	3,3	3,94	3,94	3,73	3,65	3,85	3,6	3,54	3,93	3,92	2.2	0.5 to 3.7<br><0.001	1	[2012-2013]<br>12.2<br>(3.0 to 21.8)<br>0.002	[2013-2021]<br>-0.2<br>(-3.7 to 1.7)<br>0.60
France	7,36	7,25	6,66	6,27	5,86	5,98	6,32	6,15	6,4	6,17	-2.2	-2.8 to -1.6<br><0.001	1	[2012-2016]<br>-5.6<br>(-7.8 to -4.1)<br><0.001	[2016-2021]<br>1.3<br>(-0.2 to 3.7)<br>0.09
Croatia	7,66	6,34	8,02	8,31	7,22	6,9	7,09	5,26	5,51	5,6	-3.6	-6.5 to -0.6<br>0.02	0	-	-
Italy	2,79	2,87	2,74	2,57	2,39	2,5	2,58	2,39	2,3	2,5	-1.5	-3.4 to 0.5<br>0.12	0	-	-
Cyprus	0,38	1,13	2,42	2,03	1,12	1,27	1,59	0,89	1,64	1,98	5.8	-4.1 to 16.9<br>0.28	0	-	-
Latvia	6,4	5,47	6,15	7	6,62	5,97	3,4	5,56	4,74	3,32	-5.0	-10.7 to 1.2<br>0.10	0	-	-
Lithuania	10,06	11,7	9,38	10,09	7,84	9,12	8,31	7,18	8,02	7,51	-4.4	-5.8 to -2.9	0	-	-
Luxembourg	4,69	6,53	6,22	8,43	4,75	5,16	5,71	7,03	4,43	3,15	-4.5	-9.1 to 0.2<br>0.06	0	-	-
Hungary	10,22	9,28	8,25	8,78	8,21	7,22	6,97	7,2	7,2	6,51	-4.6	-5.6 to -3.6<br><0.001	0	-	-
Malta	2,87	0,4	2,36	3,2	1,69	1,97	0,85	0,38	2,72	3,88	0.4	-14.8 to 18.3<br>0.97	0	-	-
Netherlands	6,83	6,52	6,94	6,93	7,12	7,04	7,47	6,58	6,66	6,36	-0.2	-1.0 to 0.6	1	[2012-2018]	[2018-2021]

												0.68		1.9 (0.9 to 3.7) <0.001	-4.8 (-9.6 to -1.6) <0.001
Austria	6,51	7,19	7,02	6,27	6,36	5,55	5,51	5,16	4,94	4,55	-4.1	-5.2 to -3.1 <0.001	0	-	-
Poland	4,15	4,26	4,1	3,7	3,02	3,04	2,94	3,07	3,01	3,32	-3.0	-5.2 to -1.2 <0.001	0	-	-
Portugal	3,66	4,1	5,1	5,53	3,82	4,59	4,24	3,88	3,37	3,46	-1.0	-3.9 to 2.0 0.46	1	[2012-2015] 8.1 (1.0 o 18.9) 0.01	[2016-2021] -6.6 (-3.9 to 2.0) 0.46
Romania	3,42	3,6	3,16	3,32	3,06	3,22	3,11	2,4	2,38	2,59	-4.2	-6.8 to -1.6 <0.001	0	-	-
Slovenia	7,48	8,25	5,88	8,36	7,12	7,42	7,21	7,78	6,28	8,26	-0.5	-3.2 to 2.4 0.73	0	-	-
Slovakia	2,65	3,12	2,97	2,98	2,15	2,41	2,76	2,01	1,78	2,36	-3.1	-8.2 to 2.6 0.24	0	-	-
Finland	7,79	8,09	7,01	6,2	6,06	7,67	6,82	6,51	6,85	8,03	-0.5	-4.2 to 3.3 0.73	0	-	-
Sweden	7,16	7,69	7,62	6,85	7,14	7,01	7,71	7,99	6,73	6,94	-0.1	-1.9 to 1.8 0.95	0	-	-
Iceland	7,15	9,72	6,93	7,51	2,93	1,25	3,05	4,66	8,53	6,68	-1.4	-13.7 to 12.6 0.83	0	-	-
Liechtenstein	9,23	9	15	12	20,87	13	5,38	15,71	9,44	13	5.2	-3.9 to 15.2 0.27	0	-	-
Norway	6,01	7,18	5,83	7,53	7,78	7,45	7,73	7	6,51	6,52	0.5	-2.9 to 4.1 0.75	0	-	-
Switzerland	6,93	6,94	6,61	6,54	6	6,29	6,56	6,24	6,23	6,36	-1.1	1.7 to -0.4 <0.001	0	-	-
Serbia	7,96	7,58	7,19	7,06	6,68	6,27	5,99	6,29	5,58	5,61	-4.7	-5.3 to -3.7 <0.001	0	-	-

AAMR: Age-adjusted mortality rate, expressed as deaths per 100.000 population. AAPC: Average Annual percent change; APC: Annual percent change.

Table S4. Numbers of suicide-related deaths among European countries.

	2012	2013	2014	2015	2016	2017	2018	2019	2020	2021
Belgium	1.550	1.410	1.405	1.384	1.426	1.273	1.367	1.313	1.269	1.216
Bulgaria	649	546	522	530	491	505	495	431	449	411
Czechia	1.393	1.323	1.223	1.120	1.094	1.127	1.065	952	972	956
Denmark	455	428	432	383	394	380	383	389	391	354
Germany	6.779	6.811	6.928	6.725	6.560	6.177	6.215	5.954	6.032	5.803
Estonia	190	171	194	169	143	175	145	153	149	142
Ireland	494	448	453	394	398	355	404	366	436	419
Greece	424	467	453	421	379	396	435	406	358	354
Spain	2.588	2.872	2.840	2.567	2.609	2.620	2.594	2.651	2.781	2.815
France	7.149	7.181	6.475	6.683	6.189	5.981	6.294	6.373	6.324	6.403
Croatia	600	549	564	553	517	477	521	433	422	426
Italy	3.271	3.280	3.132	3.001	2.916	2.954	2.835	2.822	2.698	2.892
Cyprus	35	41	37	35	31	31	39	31	31	21
Latvia	391	337	336	325	306	295	263	253	245	211
Lithuania	823	964	809	773	706	650	555	539	498	467
Luxembourg	38	33	57	53	35	41	38	51	52	26
Hungary	1.741	1.552	1.468	1.327	1.240	1.179	1.142	1.062	1.191	1.077
Malta	22	17	25	33	20	20	21	20	20	31
Netherlands	1.346	1.447	1.395	1.389	1.380	1.450	1.337	1.345	1.322	1.386
Austria	936	937	964	907	864	853	824	769	739	729
Poland	5.467	5.198	4.999	4.671	4.152	4.054	3.981	4.054	3.963	4.054
Portugal	772	762	847	751	671	746	692	675	635	644
Romania	2.268	2.165	1.960	1.961	1.719	1.710	1.688	1.518	1.523	1.545
Slovenia	348	363	317	334	285	302	248	277	259	308
Slovakia	538	575	490	427	365	337	361	314	312	335
Finland	610	619	556	518	577	583	565	509	519	504
Sweden	774	847	784	808	788	820	883	879	844	855
Iceland	25	31	29	28	34	33	32	34	34	26
Liechtenstein	3	3	4	1	6	6	8	10	5	1
Norway	385	407	410	432	482	469	522	493	498	500
Switzerland	786	786	768	807	777	810	748	733	699	728
Serbia	945	921	832	766	694	744	688	682	650	646

Table S5. Numbers of suicide-related deaths in men among European countries.

	2012	2013	2014	2015	2016	2017	2018	2019	2020	2021
Belgium	1.184	1.076	1.056	1.034	1.073	970	1.034	1.013	965	900
Bulgaria	520	451	421	423	386	398	396	359	365	328
Czechia	1.202	1.126	1.032	952	898	936	916	789	817	785
Denmark	365	337	351	298	317	297	308	311	297	282
Germany	5.344	5.374	5.510	5.202	5.170	4.855	4.888	4.690	4.779	4.542
Estonia	167	148	164	144	125	145	122	133	131	126
Ireland	416	369	377	316	325	286	316	283	320	337
Greece	347	371	365	336	310	322	345	344	290	299
Spain	2.016	2.218	2.166	1.956	1.987	1.960	1.958	2.041	2.118	2.169
France	5.621	5.643	5.064	5.324	4.939	4.745	4.969	5.046	4.963	5.064
Croatia	480	450	433	428	406	370	408	345	333	349
Italy	2.582	2.568	2.429	2.337	2.315	2.322	2.181	2.223	2.129	2.262
Cyprus	33	36	27	28	26	27	33	29	24	13
Latvia	344	289	283	271	260	253	237	212	207	189
Lithuania	691	817	697	651	611	540	463	466	409	379
Luxembourg	29	20	45	41	30	30	28	36	41	22
Hungary	1.440	1.282	1.209	1.078	1.023	960	943	847	974	905
Malta	16	17	20	27	17	17	19	19	14	25
Netherlands	967	1.089	1.028	1.017	1.020	1.054	929	975	936	1.013
Austria	757	717	756	723	665	683	677	602	582	591
Poland	4.849	4.556	4.367	4.091	3.669	3.550	3.525	3.561	3.471	3.540
Portugal	623	596	649	539	533	556	527	521	508	511
Romania	1.975	1.874	1.706	1.693	1.477	1.449	1.444	1.335	1.323	1.338
Slovenia	291	300	270	271	238	254	195	223	212	249
Slovakia	481	503	428	365	312	278	310	272	281	288
Finland	497	511	455	438	483	485	469	420	419	388
Sweden	601	646	574	624	590	629	680	675	648	653
Iceland	20	26	23	26	31	31	28	28	26	20
Liechtenstein	2	3	4	1	2	6	7	7	3	1
Norway	305	302	320	320	345	335	390	373	388	383
Switzerland	588	593	586	615	593	610	543	552	518	533
Serbia	727	701	652	593	526	582	540	527	509	511

Table S6. Numbers of suicide-related deaths in women among European countries.

	2012	2013	2014	2015	2016	2017	2018	2019	2020	2021
Belgium	366	334	349	350	353	303	333	300	304	316
Bulgaria	129	95	101	107	105	107	99	72	84	83
Czechia	191	197	191	168	196	191	149	163	155	171
Denmark	90	91	81	85	77	83	75	78	94	72
Germany	1.435	1.437	1.418	1.523	1.390	1.322	1.327	1.264	1.253	1.261
Estonia	23	23	30	25	18	30	23	20	18	16
Ireland	78	79	76	78	73	69	88	83	116	82
Greece	77	96	88	85	69	74	90	62	68	55
Spain	572	654	674	611	622	660	636	610	663	646
France	1.528	1.538	1.411	1.359	1.250	1.236	1.325	1.327	1.361	1.339
Croatia	120	99	131	125	111	107	113	88	89	77
Italy	689	712	703	664	601	632	654	599	569	630
Cyprus	2	5	10	7	5	4	6	2	7	8
Latvia	47	48	53	54	46	42	26	41	38	22
Lithuania	132	147	112	122	95	110	92	73	89	88
Luxembourg	9	13	12	12	5	11	10	15	11	4
Hungary	301	270	259	249	217	219	199	215	217	172
Malta	6	0	5	6	3	3	2	1	6	6
Netherlands	379	358	367	372	360	396	408	370	386	373
Austria	179	220	208	184	199	170	147	167	157	138
Poland	618	642	632	580	483	504	456	493	492	514
Portugal	149	166	198	212	138	190	165	154	127	133
Romania	293	291	254	268	242	261	244	183	200	207
Slovenia	57	63	47	63	47	48	53	54	47	59
Slovakia	57	72	62	62	53	59	51	42	31	47
Finland	113	108	101	80	94	98	96	89	100	116
Sweden	173	201	210	184	198	191	203	204	196	202
Iceland	5	5	6	2	3	2	4	6	8	6
Liechtenstein	1	0	0	0	4	0	1	3	2	0
Norway	80	105	90	112	137	134	132	120	110	117
Switzerland	198	193	182	192	184	200	205	181	181	195
Serbia	218	220	180	173	168	162	148	155	141	135

Table S7. Suicide-related age-adjusted mortality in European subjects aged less than 65 years in different European countries, 2012–2020.

	2012	2013	2014	2015	2016	2017	2018	2019	2020	2021	AAPC	95% CI p	Joinpoints	APC Period 1 [Years]; APC (95% CI)p	APC Period 2 [Years]; APC (95% CI)p
Belgium	17,22	15,97	16,04	15,61	15,72	14,42	14,63	14,26	13,84	12,74	-2.7	-3.0 to -2.3 <0.001	0	-	-
Bulgaria	8,76	7,31	6,72	7,12	6,64	6,65	6,5	5,97	5,96	5,81	-4.1	-5.3 to -2.2 <0.001	0	-	-
Czechia	14,4	13,92	12,61	11,54	11,05	11,91	11,11	9,53	9,88	9,79	-4.3	-5.6 to -3.1 <0.001	0	-	-
Denmark	10,58	9,54	10,3	8,7	8,7	8,51	8,5	8,38	8,5	7,36	-3.1	-4.8 to -1.4 <0.001	0	-	-
Germany	9,45	9,48	9,52	9,26	9,03	8,31	8,3	7,94	8,05	7,77	-2.5	-3.7 to -1.4 <0.001	0	-	-
Estonia	15,3	13,35	16,94	14,12	12,5	15,8	11,69	12,84	14,81	11,91	-1.9	-6.4 to 2.6 0..33	0	-	-
Ireland	13,36	11,54	11,41	9,92	10,32	8,78	9,83	8,64	10,29	9,65	-3.0	-5.4 to -0.7 0.01	0	-	-
Greece	4,04	4,51	4,5	4,23	3,79	4,02	4,28	4,06	3,59	3,5	-2.0	-4.3 to 0.4 0.10	0	-	-
Spain	6,03	6,68	6,8	6,12	6	6,18	6	6,2	6,56	6,79	0.3	-1.5 to 2.0 0.74	0	-	-
France	13,45	13,33	12,08	12,13	11,36	10,98	11,61	11,59	11,57	11,3	-1.9	-2.6 to -1.0 <0.001	0	-	-
Croatia	14,26	13,41	13,68	13,09	12,14	11,77	12,35	10,19	9,99	10,31	-4.0	-5.5 to -2.4 <0.001	0	-	-
Italy	5,64	5,59	5,31	5,04	4,93	4,95	4,8	4,78	4,64	4,94	-2.0	-2.5 to -1.4 <0.001	1	[2012-2016] -3.7 (-6.7 to -2.4) <0.001	[2016-2021] -0.5 (-1.6 to 2.4) 0.66
Cyprus	4,54	4,63	4,46	4,85	3,98	4,3	4,98	4,35	3,99	2,58	-5.4	-8.5 to -1.9 0.01	1	[2012-2019] 0.2 (-2.6 to 8.5) 0.69	[2019-2021] -22.6 (-35.0 to -7.8) <0.001
Latvia	20,59	18,3	18,06	18,02	17,07	16,6	14,32	14,68	14,21	12,12	-4.9	-6.0 to -3.8 <0.001	0	-	-
Lithuania	29,75	35,79	29,6	28,5	26,67	24,19	20,96	20,14	18,49	16,62	-7.5	-9.2 to -5.7 <0.001	0	-	-
Luxembourg	9,15	5,96	11,02	10,64	7,5	9,27	7,73	10,41	8,54	4,84	-2.5	-9.8 to 5.1 0.48	0	-	-
Hungary	19,92	17,85	16,52	15,1	13,88	13,08	12,71	12,31	12,83	12,15	-5.1	-5.5 to -4.6 <0.001	0	-	-
Malta	5,61	5,88	7,34	8,53	6,32	5,11	4,14	4,13	4,66	7,51	-2.5	-11.4 to 6.9 0.50	0	-	-

Netherlands	10,43	10,93	10,99	10,68	10,46	10,82	10,17	9,89	9,84	9,98	-0.8	-1.6 to -0.2<br>0.01	1	[2012-2014]<br>2.1<br>(-1.3 to 5.4)<br>0.34	[2014-2021]<br>-1.6<br>(-4.5 to -0.5]<br>0.02
Austria	11,45	11,06	11,63	10,8	10,39	10,02	9,77	9,18	8,45	8,29	-3.4	-4.1 to -2.8<br><0.001	0	-	-
Poland	16,76	16,23	15,54	14,16	11,96	11,6	11,46	11,68	11,67	11,87	-4.3	-5.1 to -3.5<br><0.001	0	-	-
Portugal	6,99	6,95	8,4	7,28	6,62	7,37	6,71	6,61	6,39	6,31	-1.1	-2.9 to 0.3<br>0.11	0	-	-
Romania	12,12	11,51	10,21	10,31	9,05	9,04	8,76	8,02	7,98	8,25	-4.1	-5.1 to -3.5<br><0.001	0	-	-
Slovenia	17,29	17,86	15,12	15,97	14,42	15,03	12,73	13,23	11,44	14,97	-3.4	-5.9 to -0.9<br>0.005	0	-	-
Slovakia	10,69	11,24	9,67	8,61	7,03	6,62	6,95	5,97	5,94	6,47	-6.9	-9.7 to -4.3<br><0.001	0	-	-
Finland	16,14	16,13	14,38	13,28	13,82	14,76	13,8	13,36	13,17	13,09	-2.3	-3.3 to -1.3<br><0.001	1	[2012-2015]<br>-5.3<br>(-10.0 to -2.4)<br><0.001	[2016-2021]<br>-0.8<br>(-2.1 to 3.3)<br>0.57
Sweden	11,46	11,84	11,04	11,33	10,77	10,8	11,97	11,95	11,33	11,29	0.1	-1.7 to 1.8<br>0.94	0	-	-
Iceland	12,12	13,2	12,03	13,73	13,32	10,57	10,01	11,92	12,28	11,07	-1.5	-4.6 to 1.6<br>0.29	0	-	-
Liechtenstein	11,97	9,29	5,96	3,09	12,93	17,56	18,28	18,38	6,09	7	5.2	-9.4 to 22.1<br>0.53	0	-	-
Norway	10,8	11,3	10,72	11,23	12,29	11,6	13,02	12,41	11,55	12,15	1.4	-0.4 to 3.2<br>0.11	0	-	-
Switzerland	10,9	11,16	10,12	10,58	10,02	10,17	9,66	9,55	9,19	9,32	-2.0	-2.8 to -1.2<br><0.001	0	-	-
Serbia	12,33	12,41	10,78	10,48	9,44	9,92	9,2	9,58	8,72	8,53	-4.1	-5.2 to -2.9<br><0.001	0	-	-

AAMR: Age-adjusted mortality rate, expressed as deaths per 100.000 population. AAPC: Average Annual percent change; APC: Annual percent change.

Table S8. Suicide-related age-adjusted mortality in European subjects aged over 65 years in different European countries, 2012–2020.

	2012	2013	2014	2015	2016	2017	2018	2019	2020	2021	AAPC	95% CI<br>P	Joinpoints	APC Period 1 [Years]; APC (95% CI)p	APC Period 2 [Years]; APC (95% CI)p
Belgium	25,24	22,79	22,46	21,93	22,75	19,66	21,69	19,48	21,14	20,95	-1.9	-3.3 to -0.4<br><0.001	0	-	-
Bulgaria	25,9	20,02	23,11	18,51	19,91	22,71	18,44	16,43	16,62	16,15	-4.2	-7.4 to -1.3<br>0.006	0	-	-
Czechia	22,46	20,39	21,6	20,31	18,72	18,38	19,87	18,23	18,93	19,28	-1.6	-2.9 to -0.4<br>0.009	0	-	-
Denmark	19	18,49	18,52	16,48	16,32	18,44	17,92	19,59	16,33	18,14	-0.4	-1.9 to 1.1<br>0.63	0	-	-
Germany	21,31	21,89	21,91	21,68	20,65	19,73	20,18	19,45	19,66	20,66	-0.9	-1.8 to -0.2<br>0.008	0	-	-
Estonia	31,21	31,86	23,95	23,2	21,8	23,08	27,87	23,36	22,39	22,69	-3.5	-5.7 to -1.1<br>0.004	1	[2012-2015]<br>-10.5<br>(-21.2 to -4.4)<br>0.002	[2015-2021]<br>0.2<br>(-2.3 to 10.3)<br>0.73
Ireland	7,02	9,12	8,25	7,7	5,43	6,76	7,68	6,22	6,87	7,93	-1.3	-5.5 to 3.2<br>0.54	0	-	-
Greece	6,02	5,82	7,04	6,57	6,39	6,57	7,57	6,97	5,86	6,84	1.0	-1.1 to 3.0<br>0.36	0	-	-
Spain	13,25	14,14	13,83	13,24	13,2	13	12,05	12,29	13,38	12,67	-1.0	-2.5 to 0.5<br>0.16	0	-	-
France	25,03	24,38	22,4	22,36	20,83	20,09	21,19	20,69	21,49	21,63	-1.6	-2.2 to -0.9<br><0.001	1	[2012 to 2017]<br>-4.2<br>(-6.9 to -2.9)<br><0.001	[2017-2021]<br>1.8<br>(-0.1 to 6.21)<br>0.07
Croatia	34,63	27,6	29,9	34,81	31,9	27,31	30,82	29,77	26,38	29,54	-1.4	-3.9 to 1.2<br>0.26	0	-	-
Italy	10,94	10,93	10,51	10,33	9,66	10,07	9,49	9,09	9,62	9,83	-1.3	-2.0 to -08<br><0.001	0	-	-
Cyprus	0,86	7,51	4,65	2,82	3,49	3,04	2,87	4,34	1,26	3,08	4.3	-24.8 to 35.4<br>0.79	1	[2012-2019]<br>-2.6<br>(-4.2 to -1.9)<br><0.001	[2019-2021]<br>3.4<br>(-0.7 to 5.9)<br>0.14
Latvia	26,92	22,14	22,67	24,78	25,31	23,66	20,73	16,52	21,31	18,59	-3.4	-6.7 to -0.2<br>0.04	0	-	-
Lithuania	34,57	37,47	39,44	37,63	34,37	33,46	36,44	34,26	32,84	31,43	-1.0	-2.3 to -0.1<br>0.03	1	[2012-2014]<br>4.8<br>(-0.8 to 10.3)<br>0.14	[2014-2021]<br>-2.7<br>(-6.5 to -1.7)<br>0.01
Luxembourg	17,02	23,07	23,03	27,37	17,13	9,83	14,28	16,12	16	11,43	-6.1	-11.8 to -0.1	1	[2012-2014]	[2014-2021]

												0.04		4.8<br>(-0.8 to 10.3)<br>0.14	-2.7<br>(-6.5 to -1.7)<br>0.01
Hungary	41,54	35,23	31,38	35,26	34,86	31,44	33,9	29,43	34,7	30,28	-2.1	-4.3 to 0.2<br>0.07	0	-	-
Malta	9,65	1,46	11,48	2,67	1,22	5,72	4,01	3,21	1,17	2,8	-9.4	-27.4 to 12.5<br>0.35	0	-	-
Netherlands	11,67	12,91	11,7	13,74	14,69	12,89	12,84	12,92	13,14	12,99	0.9	-1.6 to 3.3<br>0.37	0	-	-
Austria	30,7	33,08	30,24	29,51	26,98	29,59	29,48	25,29	25,25	27	-2.3	-4.7 to 0.1<br>0.06	0	-	-
Poland	16,49	16,82	15,37	14,51	13,61	12,11	12,7	13,13	13	13,31	-2.9	-3.6 to -2.2<br><0.001	1	[2012-2017]<br>-6.2<br>(-8.2 to -4.8)<br><0.001	[2017-2021]<br>1.4<br>(-0.6 to 4.4)<br>0.17
Portugal	22,29	21,44	23,17	23,17	18,59	18,86	18,59	17,65	17,07	16,07	-3.9	-5.4 to -2.4<br><0.001	0	-	-
Romania	14,92	15,24	15,96	16,07	14,74	13,82	15,26	13,24	13,46	13,78	-1.6	-3.8 to 0.5<br>0.12	0	-	-
Slovenia	39,02	37,53	34,37	40,3	33,25	38,09	33,94	38,83	40,29	39,76	0.5	-1.3 to 2.4<br>0.59	0	-	-
Slovakia	12,87	15,5	15,17	11,59	9,34	9,75	12,05	11,08	10,87	11,79	-2.8	-7.1 to 1.6<br>0.19	0	-	-
Finland	16,13	17,29	15,27	14,23	16,07	16,03	16,89	13,92	12,06	15,08	-1.8	-4.3 to 0.7<br>0.14	0	-	-
Sweden	16,24	17,92	16,61	16,31	15,7	17,36	15,9	16,03	14,31	15,33	-1.4	-2.8 to 0.1<br>0.07	0	-	-
Iceland	9,49	33,19	22,04	10,33	8,56	5,7	11,12	8,9	14,04	4,96	-9.6	-25.8 to 9.7<br>0.25	0	-	-
Liechtenstein	20	22	27,81	21	26,55	23	27,04	52,83	54,25	25,16	-3.3	-2.1 to 4.1<br>0.56	0	-	-
Norway	9,88	11,05	12,01	13,71	11,32	11,61	12,56	12,24	13,99	11,96	2.0	-0.9 to 4.9<br>0.17	0	-	-
Serbia	37,69	34,95	36,91	33,79	30,36	32,08	30,42	28,44	28,95	29,18	-3.1	-4.1 to -2.1<br><0.001	0	-	-

AAMR: Age-adjusted mortality rate, expressed as deaths per 100.000 population. AAPC: Average Annual percent change; APC: Annual percent change.

Table S9. Suicide-related age-adjusted mortality in Europe, stratified by European subregions, exploring different trends in the general population, 2012–2020.

	Age-Adjusted Mortality Rate (per 100,000)															
	**2012**	**2013**	**2014**	**2015**	**2016**	**2017**	**2018**	**2019**	**2020**	**2021**	**AAPC**	**95% CI**	**p**	**Joinpoints**	**APC Period 1 [Years]; APC (95% CI); p**	**APC Period 2 [Years]; APC (95% CI); p**
										Sex						
**Men**																
North	18.8	21.0	19.4	18.3	19.4	18.4	18.8	18.5	17.6	16.8	-1.3	-2.5 to 0.1	0.06	0	-	-
West	27.2	25.3	25.2	22.9	22.0	21.1	20.9	20.5	21.1	20.9	-2.9	-3.9 to -1.9	<0.001	0		
East	33.8	33.7	31.6	29.5	27.7	27.1	25.5	23.5	24.2	22.2	-4.1	-4.8 to -3.5	<0.001	1	[2012-2013] -0.5 (-4.0 to 3.0) p = 0.53	[2013-2021] -4.9 (-7.1 to -4.4) p < 0.001
South	18.6	17.9	17.5	172.	16.0	15.5	15.2	15.1	15.0	14.2	-2.5	-3.7 to -1.3	<0.001	0	-	-
**Women**																
North	7.3	7.7	8.0	7.0	6.2	6.0	7.6	6.8	6.5	6.8	-1.3	-2.5 to 0.1	0.06	0	-	-
West	7.1	7.0	6.7	6.8	6.7	7.1	5.5	6.6	6.1	5.0	-2.9	-3.9 to -1.9	<0.001	0	-	-
East	5.9	5.8	5.7	5.6	5.5	5.0	4.7	4.6	4.5	4.3	-4.1	-4.8 to -3.5	<0.001	1	[2012-2013] -0.5 (-4.0 to 3.0) p=0.53	[2013-2021] -4.9 (-7.1 9to -4.4) p<0.001
South	4.3	4.1	4.0	3.9	4.0	3.9	3.8	3.6	3.5	3.3	-2.5	-3.7 to -1.3	<0.001	0	-	-
										Age						
**<65 years**																
North	12.5	13.1	12.0	12.6	12.7	11.6	11.7	12.3	12.1	11.7	-0.8	-1.9 to 0.3	0.13	0	-	-
West	14.1	13.7	13.1	12.2	12.2	12.1	11.8	11.7	10.5	9.3	-4.2	-4.9 to -3.4	<0.001	0	-	-
East	16.5	16.6	15.6	14.6	13.4	13.6	11.9	11.5	11.5	10.4	-5.2	-6.4 to -4.1	<0.001	0	-	-
South	8.5	8.6	8.4	8.4	7.6	7.5	7.3	7.2	6.6	6.2	-2.8	-4.2 to -1.4	<0.001	0	-	-
**≥65 years**																
North	13.9	13.8	13.4	13.6	12.9	12.6	12.4	12.0	11.9	11.7	-2.8	-6.6 to 1.1	0.13	0	-	-
West	20.8	21.2	22.7	20.6	21.1	17.8	21.2	22.4	22.8	20.0	0.1	-2.0 to 2.2	0.99	0	-	-
East	19.3	17.9	19	18.6	16.4	17.1	16.4	16.7	16.4	16.0	-2.7	-3.4 to -2.0	<0.001	0	-	-
South	19.3	18.6	17.9	18.4	16.7	16.7	16.5	16.4	16.2	16.4	-1.9	-3.2 to -0.5	0.009	0	-	-

AAMR: Age-adjusted mortality rate, expressed as deaths per 100.000 population. AAPC: Average Annual percent change; APC: Annual percent change. Southern Europe: Greece, Spain, Italy, Cyprus, Malta, Portugal, Slovenia and Serbia; Northern Europe: Finland, Sweden, Iceland and Norway; Western Europe: Belgium, Denmark, Germany Ireland, France, France, Luxembourg, Hungary, Netherlands, Austria, Poland, Liechtenstein, and Switzerland; Eastern Europe: Bulgaria, Czech Republic, Estonia, Latvia, Lithuania and Slovakia.

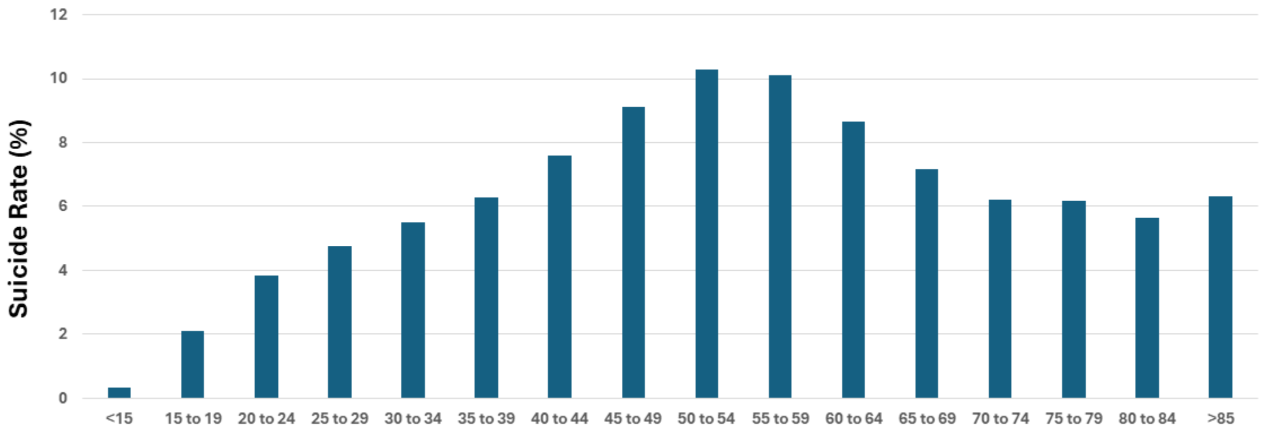

Figure S1. Suicide-related mortality rates in Europe by age group, 2012–2021.
